# A Comparative Study on Maternal and Perinatal Outcomes in Oral Hydration Therapy With Oral Rehydration Solution (ORS) Versus No Hydration Therapy in Term Pregnancies With Isolated Oligohydramnios

**DOI:** 10.7759/cureus.82016

**Published:** 2025-04-10

**Authors:** Annapurna S Hadalageri, Rajasri G Yaliwal, Mallanagouda Patil, Shobha Shirgur, Shreedevi Kori

**Affiliations:** 1 Obstetrics, BLDE (Deemed to be University) Shri B. M. Patil Hospital, Medical College and Research Center, Vijayapura, IND; 2 Obstetrics and Gynaecology, BLDE (Deemed to be University) Shri B. M. Patil Hospital, Medical College and Research Center, Vijayapura, IND; 3 Pediatrics, BLDE (Deemed to be University) Shri B. M. Patil Hospital, Medical College and Research Center, Vijayapura, IND

**Keywords:** amniotic fluid index, feto-maternal outcome, oligohydramnios, oral rehydration therapy, umbilical artery

## Abstract

Introduction

Oligohydramnios, a condition characterized by reduced amniotic fluid volume, is associated with adverse maternal and perinatal outcomes, including increased rates of cesarean section, fetal distress, and neonatal intensive care unit (NICU) admissions. Maternal hydration therapy, particularly oral rehydration solution (ORS), has been proposed as a simple and cost-effective intervention to improve the amniotic fluid index (AFI) and perinatal outcomes in pregnancies complicated by isolated oligohydramnios. This study aimed to evaluate the effectiveness of ORS therapy compared to no hydration in term pregnancies with isolated oligohydramnios.

Materials and methods

A randomized controlled trial was conducted at Shri B.M. Patil Medical College and Hospital, India. A total of 60 pregnant women with singleton term pregnancies (37-40 weeks) diagnosed with isolated oligohydramnios (AFI 5-8 cm) were randomized into two groups: Group 1 (ORS Therapy, n=30) received ORS (2 liters/day for 3 days), while Group 2 (no hydration, n=30) followed a routine dietary intake. AFI was measured at baseline and after 72 hours. Secondary outcomes included mode of delivery, birth weight, NICU admissions, Apgar scores, and umbilical artery blood gas analysis. Data were analyzed using IBM SPSS Statistics v. 26 (IBM Corp., Armonk, NY, US), with a p-value < 0.05 considered statistically significant.

Results

ORS therapy significantly increased AFI after 72 hours (p=0.026). Spontaneous vaginal delivery was higher in the ORS group (50%) compared to the no hydration group (33.3%), while cesarean section rates were lower (23.3% vs. 46.7%). NICU admissions were significantly reduced in the ORS group (23.3% vs. 50%; p=0.0321), and birth weight <2.5 kg was less frequent (13.3% vs. 36.7%; p=0.0368). Umbilical artery blood gas analysis showed better fetal oxygenation in the ORS group (p<0.05).

Conclusion

ORS therapy is an effective, non-invasive intervention for increasing AFI, reducing cesarean rates, and improving neonatal outcomes in term pregnancies with isolated oligohydramnios. Given its affordability and safety, ORS therapy should be considered a first-line treatment before invasive interventions. Further large-scale trials are needed to establish standardized protocols for its use in obstetric practice.

## Introduction

Oligohydramnios is a significant obstetric condition that can lead to fetal distress, umbilical cord compression, increased cesarean delivery rates, and adverse neonatal outcomes [1]. It can result from maternal conditions, such as dehydration, hypertensive disorders, or diabetes, as well as fetal and placental factors like growth restriction, congenital renal anomalies, or placental insufficiency [2,3]. However, isolated oligohydramnios, where there are no identifiable maternal or fetal complications, remain a concern due to their association with higher rates of labor induction, abnormal fetal heart patterns, and neonatal intensive care unit (NICU) admissions [4].

Maternal hydration therapy has been explored as a non-invasive, cost-effective method to increase amniotic fluid volume [5,6]. The underlying physiological mechanism suggests that increased maternal plasma volume enhances placental perfusion and fetal urine output, contributing to improved AFI [7]. While intravenous hydration therapy has been studied, there is growing interest in oral hydration therapy, particularly oral rehydration solution (ORS), which is widely used for fluid and electrolyte replenishment in various medical conditions [8]. Some studies indicate that maternal oral hydration can significantly improve AFI within 48-72 hours, reducing the need for invasive interventions like amnioinfusion or early labor induction [3,5]. However, limited data exist on the comparative efficacy of ORS therapy versus no hydration therapy in cases of isolated oligohydramnios at term [9,10].

This study aims to evaluate the effect of ORS therapy on AFI, mode of delivery, neonatal outcomes (birth weight, NICU admissions, Apgar scores), and maternal complications in term pregnancies with isolated oligohydramnios. By establishing ORS therapy as a potential first-line intervention, this research could provide a simple, accessible, and effective alternative to invasive procedures, particularly in low-resource settings where amnioinfusion or intensive maternal-fetal monitoring may not always be feasible. If proven effective, ORS therapy could be integrated into standard prenatal care, offering a safe and economical approach to managing isolated oligohydramnios, ultimately improving maternal and neonatal outcomes.

## Materials and methods

This study was conducted as a randomized controlled trial at the Department of Obstetrics and Gynecology, Shri B. M. Patil Medical College and Hospital, Vijayapura, Karnataka, India. Pregnant women presenting to the antenatal outpatient department and labor ward were screened for eligibility based on predefined inclusion and exclusion criteria. A total of 1089 pregnant women with a gestational age of 37-40 weeks were screened, of whom 1029 were excluded based on exclusion criteria. The remaining 60 consenting women were randomized into two groups using a computer-generated randomization program: Group 1 (ORS therapy, n = 30) received oral hydration therapy with ORS (2 liters daily for 3 days), while Group 2 (no hydration, n = 30) did not receive additional hydration therapy beyond routine dietary intake.

The study included pregnant women with singleton pregnancies in cephalic presentation, confirmed gestational age between 37-40 weeks by ultrasound and last menstrual period (LMP), and oligohydramnios (AFI < 5 cm) (4 pocket measurement) without other maternal or fetal complications. Women with hypertensive disorders of pregnancy, premature rupture of membranes, gestational diabetes mellitus, intrauterine growth restriction, congenital malformations, intrauterine fetal demise, or non-reassuring fetal status were excluded. Informed consent was obtained from all eligible participants before enrolment.

Participants in Group 1 received ORS therapy in addition to their routine diet, while Group 2 followed their usual dietary intake without any specific hydration intervention. A baseline ultrasound scan was performed for all participants to assess AFI and fetal well-being. AFI measurements were repeated after 72 hours to assess changes following the intervention. The mode of delivery, maternal complications, and neonatal outcomes were recorded for both groups. The decision for expectant management or delivery was made based on standard clinical guidelines.

The primary outcome measure was the change in AFI after hydration therapy. Secondary outcomes included mode of delivery (spontaneous vaginal delivery, induced labor, cesarean section), neonatal outcomes, such as birth weight, Apgar scores, NICU admissions, and meconium-stained liquor, as well as maternal complications like postpartum hemorrhage, hypertensive disorders, and infections. Additionally, umbilical artery blood gas analysis (pH, pO₂, pCO₂, bicarbonate levels, and base deficit) was performed to assess fetal acid-base status.

**Figure 1:**
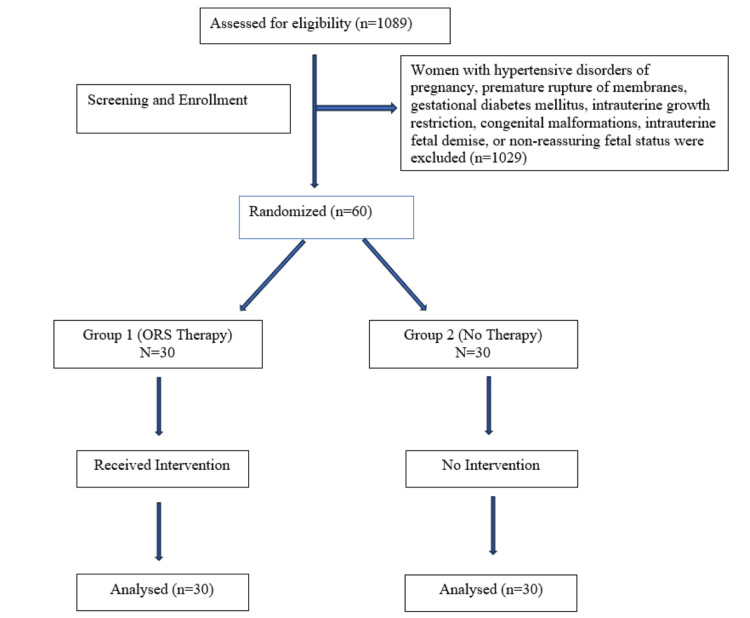
CONSORT diagram CONSORT: Consolidated Standards of Reporting Trials

Data were recorded in Microsoft Excel (Microsoft Corporation, Redmond, WA, US) and analyzed using IBM SPSS Statistics v. 26 (IBM Corp., Armonk, NY, US). Normally distributed variables were analyzed using the independent t-test. Categorical variables were compared using the chi-square test, and a p-value of < 0.05 was considered statistically significant. Ethical approval for the study was obtained from the Institutional Ethics Committee (IEC-1120/2024-25) of BLDE, Vijayapura, and the study was registered with the Clinical Trials Registry of India (CTRI No: CTRI/2024/10/075111). Written informed consent was obtained from all participants, ensuring confidentiality and voluntary participation (Figure 1).

## Results

The age distribution of participants was comparable between the two groups. In Group 1 (ORS therapy), 23.3% of participants were younger than 20 years, 50% were between 21 and 25 years, 20% were between 26 and 30 years, and 6.7% were older than 30 years. Similarly, in Group 2 (no hydration), 16.7% were younger than 20 years, 56.7% were between 21 and 25 years, 13.3% were between 26 and 30 years, and 13.3% were older than 30 years. The distribution did not show a significant difference between the groups (p=0.6765). The majority of participants in both groups were between 37 and 38 weeks of gestation (63.3% in Group 1 and 73.3% in Group 2), with no significant difference in gestational age distribution (p=0.5415). Obstetric history was also similar between the groups, with 46.7% of primigravida in Group 1 and 43.3% in Group 2 (p=0.7952) (Table 1). Regarding delivery outcomes, spontaneous vaginal delivery was more frequent in Group 1 (50%) compared to Group 2 (33.3%), while cesarean section rates were higher in Group 2 (46.7%) than in Group 1 (23.3%). However, this difference was not statistically significant (p=0.1637). Postpartum hemorrhage occurred in 6.7% of Group 1 and 13.3% of Group 2, but this difference was not significant (p=0.3894). Birth weight <2.5 kg was more common in Group 2 (36.7%) than in Group 1 (13.3%), showing a significant association (p=0.0368). NICU admissions were also significantly higher in Group 2 (50%) as compared to Group 1 (23.3%) (p=0.0321) (Table 2).

**Table 1:** Comparison of AFI at baseline and 72 hours between groups Frequency and percentages were calculated. The chi-square test was done, and a p-value of less than 0.05 was considered significant. AFI: amniotic fluid index

AFI	Group 1 (ORS Therapy)	Group 2 (No Hydration)	Chi-Square Value	p-Value
Baseline				
5-6 cm	5 (16.7%)	6 (20%)	0.1231	0.9402
6-7 cm	9 (30%)	9 (30%)		
7-8 cm	16 (53.3%)	15 (50%)		
After 72 hours				
5-6 cm	1 (3.3%)	5 (16.7%)	7.2813	0.026
6-7 cm	14 (46.7%)	19 (63.3%)		
7-8 cm	15 (50%)	6 (20%)		

**Table 2:** Comparison of demographic and obstetric characteristics between groups Frequency and percentages were calculated, and the chi-square test was done.

Variable	Group 1 (ORS Therapy)	Group 2 (No Hydration)	Chi-Square Value	p-Value
Age (Years)				
< 20 years	7 (23.3%)	5 (16.7%)	1.525	0.6765
21-25 years	15 (50%)	17 (56.7%)		
26-30 years	6 (20%)	4 (13.3%)		
> 30 years	2 (6.7%)	4 (13.3%)		
Gestational Age				
37-38 weeks	24 (63.3%)	22 (73.3%)	0.3726	0.5415
39-40 weeks	6 (20%)	8 (26.7%)		
Obstetric History				
Primigravida	14 (46.7%)	13 (43.3%)	0.0673	0.7952
Multigravida	16 (53.3%)	17 (56.7%)		

The baseline amniotic fluid index (AFI) was comparable between the two groups, with most participants having AFI between 7 and 8 cm (53.3% in Group 1 and 50% in Group 2). However, after 72 hours, a significant improvement in AFI was observed in Group 1 as compared to Group 2. In Group 1, 50% of participants had an AFI of 7-8 cm, while in Group 2, only 20% maintained this level. Additionally, fewer participants in Group 1 had AFI between 5 and 6 cm after 72 hours (3.3%) compared to Group 2 (16.7%), showing a statistically significant difference (p=0.026) (Table 1).

Regarding delivery outcomes, spontaneous vaginal delivery was more frequent in Group 1 (50%) compared to Group 2 (33.3%), while cesarean section rates were higher in Group 2 (46.7%) than in Group 1 (23.3%). Postpartum hemorrhage occurred in 6.7% of Group 1 and 13.3% of Group 2. Birth weight <2.5 kg was more common in Group 2 (36.7%) than in Group 1 (13.3%), showing a significant association (p=0.0368). NICU admissions were also significantly higher in Group 2 (50%) compared to Group 1 (23.3%) (p=0.0321) (Table 3).

**Table 3:** Comparison of maternal and neonatal outcomes between groups Frequency and percentages were calculated, and the chi-square test was done.

Outcome	Group 1 (ORS Therapy)	Group 2 (No Hydration)	Chi-Square Value	p-Value
Mode of Delivery				
Spontaneous Vaginal Delivery	15 (50.0%)	10 (33.3%)	3.619	0.1637
Induced Vaginal Delivery	8 (26.7%)	6 (20.0%)		
Cesarean Section	7 (23.3%)	14 (46.7%)		
Postpartum Hemorrhage (PPH)	2 (6.7%)	4 (13.3%)	0.7407	0.3894
Maternal Infections	0 (0%)	0 (0%)	0	1
Birth Weight <2.5 kg	4 (13.3%)	11 (36.7%)	4.3555	0.0368
Apgar Score < 7 at 1 min	0 (0%)	0 (0%)	0	1
Apgar Score <7 at 5 min	0 (0%)	0 (0%)	0	1
NICU Admission	7 (23.3%)	15 (50.0%)	4.5933	0.0321

Umbilical artery blood gas analysis revealed significant differences in PCO2, PO2, and pH between the two groups. PCO2 levels were significantly lower in Group 1 (43.30 ± 13) compared to Group 2 (49.80 ± 9.29) (p=0.0148), while PO2 levels were significantly higher in Group 1 (22.18 ± 8.88) than in Group 2 (17.78 ± 9.69) (p=0.0357). Additionally, the pH was significantly different between the groups (p=0.0126). However, HCO3 levels did not differ significantly (p=0.21636) (Table 4).

**Table 4:** Comparison of umbilical artery blood gas parameters between groups Mean and SD were calculated, and the t-test was done.

Umbilical Artery Blood Gas Analysis	Group 1 (ORS Therapy)	Group 2 (No Hydration)	t Value	p-Value
PCO2	43.30 ± 13	49.80 ± 9.29	2.2279	0.0148
PO2	22.18 ± 8.88	17.78 ± 9.69	1.8365	0.0357
HCO3	21.93 ± 1.48	21.57 ± 2.01	0.79	0.21636
pH	7.33 ± 0.14	7.39 ± 0.05	2.2947	0.0126

## Discussion

Oligohydramnios, characterized by an amniotic fluid index (AFI) of less than 5 cm, is a significant obstetric concern due to its association with increased rates of cesarean section, low birth weight, and neonatal intensive care unit (NICU) admissions. The present study aimed to evaluate the impact of ORS therapy compared to no hydration on AFI and maternal-neonatal outcomes in term pregnancies with isolated oligohydramnios. The findings demonstrated a significant improvement in AFI following ORS therapy, which aligns with existing literature suggesting that maternal hydration effectively enhances amniotic fluid volume [11].

The study observed that after 72 hours, 50% of the ORS group achieved an AFI between 7 and 8 cm, compared to only 20% in the no hydration group (p=0.026) (p=0.0084). This outcome supports findings from previous studies where maternal hydration was linked to improved AFI levels within 24 to 48 hours [12]. Casey et al. and Ghosh R et al. also reported similar findings, with mean AFI improvements following hydration interventions [13,14]. These results indicate that ORS therapy is an effective non-invasive approach to increasing AFI, potentially mitigating complications associated with oligohydramnios.

In terms of delivery outcomes, spontaneous vaginal delivery rates were higher in the ORS group (50%) compared to the no hydration group (33.3%), while cesarean section rates were lower (23.3% vs. 46.7%), although the difference was not statistically significant (p=0.1637). Previous studies, including Chaudhary et al., have similarly demonstrated that maternal hydration is associated with increased spontaneous vaginal delivery rates and reduced fetal distress [15]. Furthermore, the present study found a significantly lower incidence of low birth weight (<2.5 kg) in the ORS group (13.3%) compared to the no hydration group (36.7%) (p=0.0368), which is consistent with findings from Garmel et al. [16].

The study also examined neonatal outcomes, revealing that NICU admissions were significantly higher in the no hydration group (50%) compared to the ORS group (23.3%) (p=0.0321). This finding suggests that ORS therapy may contribute to improved perinatal outcomes, potentially by enhancing fetal oxygenation and acid-base balance. Supporting this hypothesis, umbilical artery blood gas analysis indicated significantly lower PCO2 levels (p=0.0148) and higher PO2 levels (p=0.0357) in the ORS group, reflecting better fetal oxygenation. Similar improvements in fetal well-being parameters following maternal hydration have been documented in studies by Chauahan et al. and Doi et al. [17,18].

The study's findings reinforce the growing evidence that maternal hydration, particularly using ORS, is a simple, cost-effective intervention to improve AFI and neonatal outcomes in cases of isolated oligohydramnios. While previous studies have predominantly focused on intravenous hydration, the present study highlights the efficacy of oral hydration therapy as a viable alternative. The comparable improvements in AFI between oral and intravenous hydration groups in prior studies suggest that oral rehydration is an equally effective strategy for managing mild-to-moderate oligohydramnios [12].

The strengths of this study include its randomized controlled design, objective AFI measurement, and comprehensive neonatal outcome analysis, ensuring reliability and clinical relevance. However, limitations such as the small sample size, short study duration, lack of comparison with intravenous hydration, and potential variability in maternal hydration status before the intervention should be considered. Future research should focus on larger, multicentre trials, comparisons between ORS and intravenous hydration therapy, long-term neonatal outcomes, and the impact of hydration therapy in high-risk pregnancies.

## Conclusions

This study provides strong evidence that ORS significantly improves the amniotic fluid index (AFI), reduces cesarean section rates, and enhances neonatal outcomes in term pregnancies with isolated oligohydramnios. The findings highlight ORS therapy as a simple, non-invasive, and effective intervention that can mitigate fetal distress, improve fetal oxygenation, and decrease NICU admissions. Given its affordability, ease of administration, and favorable safety profile, ORS therapy should be considered a first-line treatment before resorting to invasive procedures such as amnioinfusion or labor induction. The study underscores the critical role of maternal hydration in optimizing pregnancy outcomes, emphasizing the need for further research to establish standardized clinical guidelines. Integrating ORS therapy into routine antenatal care can help reduce perinatal complications and improve maternal-fetal health, especially in low-resource settings where access to advanced interventions is limited.
